# Outcomes of Coronary Artery Bypass Grafting in Patients With Impaired Left Ventricular Function and the Role of Preoperative Myocardial Viability

**DOI:** 10.7759/cureus.76198

**Published:** 2024-12-22

**Authors:** Muhammad Aasim, Raheela Aziz, Atta ul Mohsin, Raheel Khan, Ayesha Zahid, Muhammad Awais, Leonardo A Marquez Roa, Nauman Shaukat, Jibran Ikram

**Affiliations:** 1 Cardiac Surgery, Hayatabad Medical Complex Peshawar, Peshawar, PAK; 2 Cardiovascular Medicine, Hayatabad Medical Complex Peshawar, Peshawar, PAK; 3 Outcomes Research, Cleveland Clinic Foundation, Cleveland, USA; 4 Cardiovascular Medicine, Royal Cornwall Hospitals NHS Trust, Truro, GBR

**Keywords:** cardiac magnetic resonance (cmr) imaging, coronary artery bypass grafting (cabg), echocardiography, ischemic left ventricular dysfunction, late gadolinium enhancement (lge), myocardial viability

## Abstract

Background

Coronary artery bypass grafting (CABG) improves outcomes in patients with ischemic left ventricular (LV) dysfunction, but accurate patient selection remains critical. Late gadolinium enhancement (LGE) on cardiac magnetic resonance (CMR) imaging aids in assessing myocardial viability, a key predictor of surgical outcomes. This study aimed to evaluate the impact of myocardial viability on postoperative outcomes in patients undergoing CABG.

Methods

This was a single-center prospective analysis of clinical outcomes in 37 patients with impaired LV function (mean ejection fraction: 35.59%); myocardial viability was assessed using LGE-CMR prior to CABG. Patient demographics, perioperative details, and short-term outcomes, including in-hospital mortality and recovery metrics, were analyzed.

Results

Patients exhibited high myocardial viability (mean: 88.16%), with an average of 2.35 non-viable segments. In-hospital mortality was 5.4% (n=2), and the mean hospital stay was six days. Patients with greater viability demonstrated better recovery and fewer complications. Multivessel coronary artery disease was prevalent (94.6%, n=35), with tailored graft configurations addressing individual anatomical and disease complexities.

Conclusion

LGE-CMR is a valuable tool for predicting outcomes in ischemic LV dysfunction. Myocardial viability strongly correlates with improved surgical recovery, highlighting the importance of integrating LGE-CMR into preoperative decision-making. Further studies are required to explore the long-term impact of myocardial viability on treatment outcomes and quality of life.

## Introduction

Left ventricular (LV) function serves as a key prognostic indicator in patients with coronary artery disease (CAD). As the incidence of CAD and ischemic LV dysfunction continues to rise, it presents an increasingly significant clinical challenge [1]. Coronary artery bypass grafting (CABG) has been shown to improve LV function and yield excellent long-term outcomes in patients with ischemic LV dysfunction [2,3]. However, perioperative morbidity and mortality are significantly higher in patients with advanced three-vessel CAD and impaired LV function. In-hospital mortality rates range from 5% to 20%, with variability largely influenced by factors such as the degree of left ventricular ejection fraction (LVEF) and the severity of congestive heart failure [4-7]. Therefore, careful selection of patients with impaired LV function who are potential candidates for revascularization with CABG is crucial. A thorough assessment of LV function, comorbidities, and the severity of ischemic damage is essential to identify those who are most likely to benefit from surgery, while minimizing the risks of perioperative complications and adverse outcomes.

Gadolinium-enhanced cardiac magnetic resonance (CMR) imaging has been proposed as a superior modality for predicting the potential lack of functional recovery in chronic ischemic cardiomyopathy and is widely recognized as the gold standard for assessing ventricular function [8,9]. Late gadolinium enhancement (LGE) CMR is essential for evaluating myocardial viability, to help guide clinical decisions regarding revascularization and management in patients with ischemic heart disease. Previous studies using gadolinium-enhanced CMR have demonstrated a significant correlation between the extent of LGE and myocardial viability following revascularization [10,11]. The prognostic impact of LGE-CMR viability assessment remains incompletely understood. Several studies have shown that the presence of delayed enhancement (DE), indicating nonviable myocardium, is associated with an increased risk of adverse outcomes in patients with and without CAD. However, these studies did not evaluate whether revascularization could improve survival in these patients [12-16]. In contrast, earlier studies using nuclear imaging or stress echocardiography suggested that viable myocardium, rather than nonviable tissue, is associated with worse prognosis when patients are treated medically [17]. The aims of this study were to evaluate the outcomes of CABG in patients with impaired LV function, as assessed by echocardiography, and to investigate the role of myocardial viability, as determined by CMR. Specifically, the study sought to assess how these imaging modalities contribute to the decision-making process for proceeding with CABG and to evaluate their impact on post-surgical outcomes.

## Materials and methods

This study conducted at Hayatabad Medical Complex, Peshawar, was a single-center prospective analysis of clinical outcomes in patients with impaired LV function as assessed by preoperative transthoracic echocardiography. The patients underwent a myocardial viability assessment using CMR imaging as part of the preoperative workup for surgical revascularization. Recruitment for the study took place from January 1, 2023, to December 31, 2023. All participants demonstrated impaired LVEF on CMR. The decision to proceed with either CABG or optimal medical therapy (OMT) was made by a clinical team, based on a comprehensive review of the patients' clinical status, multimodal imaging results (including LGE), and the patients’ preferences. A total of 37 patients with impaired LV function underwent preoperative gadolinium-enhanced CMR imaging to assess myocardial viability. This group of 37 patients (comprising 36 on-pump CABGs and 1 off-pump CABG) was included in the present study. Clinical data, including patient demographics, comorbidities, and echocardiographic measurements, were collected at the time of surgery. In addition, CMR parameters, such as myocardial scar burden and total viability, were recorded. Perioperative parameters, including cardiopulmonary bypass (CPB) time, cross-clamp time, use of intra-aortic balloon pump (IABP), ventilation hour and hospital length of stay, were also documented. Short-term outcomes, such as in-hospital complications and overall survival, were closely monitored and analyzed in relation to these preoperative imaging findings. This comprehensive dataset facilitated an in-depth evaluation of how preoperative CMR and echocardiography influence both the decision-making process for proceeding with CABG and the short-term surgical outcomes.

The primary aim of this study was to evaluate the impact of left ventricular myocardial viability, as assessed by LGE-CMR, on the outcomes of coronary artery bypass grafting in patients with ischemic heart disease and impaired left ventricular function. Secondary objectives included examining the relationship between myocardial viability and key surgical outcomes such as in-hospital mortality, recovery metrics (e.g., ventilation hours, CPB time, cross-clamp time), and complications, as well as comparing LGE-CMR to other preoperative imaging modalities in relation to CABG success.

This study was approved by the Institutional Research and Ethical Committee (IREB), Hayatabad Medical Complex, Peshawar, Pakistan (approval number 1960), ensuring that all procedures were conducted in accordance with ethical standards and patient safety protocols.

Surgical technique

The surgeon was experienced in the CABG technique, having performed over 1400 procedures, including a significant number involving patients with impaired LVEF. Grafting was carried out under full CBP with cardioplegic arrest. The left internal mammary artery (LIMA) was consistently used to graft the left anterior descending artery (LAD), with no incidents of inadequate or damaged LIMA reported. For any additional grafts, a saphenous vein graft (SVG) was utilized. Additionally, two cases were performed using total arterial grafting, while one case was done off-pump.

Statistical analysis

Statistical analysis was performed using IBM SPSS Statistics, version 20 (IBM Corp., Armonk, NY). Continuous variables were expressed as means ± standard deviations (SDs), while categorical variables were presented as frequencies and percentages.

## Results

This study analyzed a cohort of 37 patients undergoing surgical intervention, revealing a demographic predominance of male (62.2%, n=23) patients (females, 37.8%, n=14); the mean age was 55.59 years (±8.92), indicating a middle-aged population. The average body mass index was 25.80 (±2.56), reflecting a range within the normal weight to overweight classification. The mean ejection fraction (EF%) was notably low at 35.59 (±3.54), suggesting significant LV impairment (Table 1).

**Table 1:** Continuous parameters

Parameter	Mean ± SD
Age	55.59 ± 8.915
Body mass index	25.80 ± 2.56
Ejection fraction (EF%)	35.59 ± 3.54

The findings highlighted a substantial burden of cardiovascular risk factors, with 48.6% (n=18) patients diagnosed with diabetes mellitus and 56.8% (n=21) suffering from hypertension. Additionally, 18.9% (n=7) of the cohort were smokers, underscoring the multifactorial nature of their cardiovascular health. Hyperlipidemia was present in 16.2% (n=6) patients, while prior renal impairment was noted in 2.7% (n=1), and a history of cerebrovascular accidents was recorded in 5.4% (n=2) of cases. The utilization of IABP support occurred in 2.7% (n=1) patients, indicating the severity of their conditions. In-hospital mortality was observed in 5.4% (n=2) of the cohort, with recent myocardial infarctions reported in 16.2% (n=6). Endarterectomy was performed in 13.5% (n=5) patients, reflecting the complexity of their vascular conditions (Table 2).

**Table 2:** Demographic and clinical characteristics

Characteristic	Number (%)
Sex	
Male	23 (62.2%)
Female	14 (37.8%)
Diabetes	18 (48.6%)
Smoking status	7 (18.9%)
Hypertension	21 (56.8%)
Prior renal impairment	1 (2.7%)
Hyperlipidemia	6 (16.2%)
Prior cerebrovascular accident	2 (5.4%)
Intra-aortic balloon pump	1 (2.7%)
In-hospital mortality	2 (5.4%)
Recent myocardial infarction	6 (16.2%)
Endarterectomy	5 (13.5%)

These results emphasize the significant prevalence of comorbidities in this patient population, which is critical for informing perioperative management strategies and optimizing surgical outcomes.

The coronary angiography findings in this study revealed a predominance of triple vessel coronary artery disease, affecting 94.6% (n=35) of the patients. Only one patient each was diagnosed with double vessel and single vessel coronary artery disease, representing 2.7% (n=1) each. Prior percutaneous coronary interventions (PCI) were noted in several patients, with five individuals (13.5%, n=5) having undergone PCI to the LAD, two (5.4%, n=2) with PCI to the right coronary artery (RCA), and one (2.7%, n=1) with PCI to the left circumflex artery. Additionally, three patients (8.1%, n=3) had a history of multivessel PCI. Regarding left main stem disease, 5.4% (n=2) had less than 50% stenosis, while 16.2% (n=6) had more than 50%, with 5.4% (n=2) showing more than 70% stenosis (Table 3).

**Table 3:** Coronary angiography findings PCI: percutaneous coronary intervention

Findings	Number (%)
Triple vessel coronary artery disease	35 (94.6%)
Double vessel coronary artery disease	1 (2.7%)
Single vessel coronary artery disease	1 (2.7%)
Prior percutaneous coronary intervention	
PCI to the left anterior descending artery	5 (13.5%)
PCI to the right coronary artery	2 (5.4%)
PCI to the left circumflex artery	1 (2.7%)
Multivessel PCI	3 (8.1%)
Left main stem disease	
Less than 50%	2 (5.4%)
More than 50%	6 (16.2%)
More than 70%	2 (5.4%)

These findings underscore the complex coronary anatomy and significant disease burden in this patient population, which are essential to take into account for guiding treatment strategies.

The analysis of grafted vessel configurations revealed a variety of surgical strategies employed during the procedures. The most common configuration involved the LIMA to the LAD, supplemented by SVGs to the posterior descending artery (PDA), obtuse marginal (OM) branches, and diagonal arteries, accounting for 24.3% (n=9) of cases. Another significant group (35.1%, n=13) utilized the LIMA to the LAD with SVGs to both the PDA and the OM1 and OM2 (sequential) branches, in addition to diagonal grafts. Additionally, there was a configuration where the LIMA was grafted to the LAD with SVGs to the posterior left ventricular (PLV) artery and PDA sequentially, alongside SVGs to the OM and diagonal arteries, which constituted 13.5% (n=5) of the cases. The configuration involving the LIMA to the LAD, SVG to the RCA, OM, and diagonal arteries was noted in another 13.5% (n=5) of patients. Two cases employed the right internal mammary artery for grafting to the OM, along with a radial artery graft to the RCA. Notably, a single graft configuration involved the LIMA to the LAD with an SVG solely to the diagonal artery that was one off-pump case (Table 4).

**Table 4:** Grafted vessel configurations details LIMA: left internal mammary artery, LAD: left anterior descending artery, SVG: saphenous vein graft, PDA: posterior descending artery, OM: obtuse marginal, PLV: posterior left ventricular artery, DIAG: diagonal arteries, RIMA: right internal mammary artery, RCA: right coronary artery

Grafted vessel configurations	Number (%)
LIMA to LAD, SVG to PDA, SVG to OM, SVG to DIAG	9 (24.3%)
LIMA to LAD, SVG to RCA, SVG to OM, SVG to DIAG	5 (13.5%)
LIMA to LAD, SVG to PDA, SVG to OM1 + OM2, SVG to DIAG	13 (35.1%)
LIMA to LAD, SVG to PLV + PDA, SVG to OM1 + OM2, SVG to DIAG	2 (5.4%)
LIMA to LAD, RIMA to OM, Radial artery to RCA	2 (5.4%)
LIMA to LAD, SVG to PLV + PDA, SVG to OM, SVG to DIAG	5 (13.5%)
LIMA to LAD, SVG to DIAG	1 (2.7%)

These findings illustrate the complexity of grafting strategies tailored to individual patient anatomy and disease severity, which are critical for optimizing surgical outcomes.

During the surgical intervention, patients received an average of 4.46 grafts (±0.75), reflecting the complexity of their conditions. The average duration of CPB was 165.62 minutes (±35.72), with a cross-clamp time averaging 97.92 minutes (±23.73). Postoperatively, patients required ventilation for an average of 8 hours (±2.71), indicating the need for close monitoring after surgery. The average hospital stay was 6 days (±0.47), suggesting a relatively stable recovery period for the cohort (Table 5).

**Table 5:** Perioperative parameters

Parameter	Mean ± SD
No. of grafts (counts)	4.46 ± 0.75
Cardiopulmonary bypass time (minutes)	165.62 ± 35.72
Cross-clamp time (minutes)	97.92 ± 23.73
Ventilation (hours)	8.00 ± 2.71
Hospital stay (days)	6.00 ± 0.47

These parameters offer valuable insights into the clinical characteristics and perioperative context of the study population, emphasizing the importance of thorough preoperative assessments and tailored surgical approaches.

The analysis of cardiac magnetic resonance imaging (CMRI) revealed important insights into myocardial viability in the patient cohort. On average, patients had 2.35 non-viable segments (±1.060), indicating the presence of myocardial regions with compromised function. In contrast, the total myocardial viability was high, averaging 88.16% (±6.842) (Table 6).

**Table 6:** Cardiac magnetic resonance imaging findings

Parameter	Mean ± SD
Number of non-viable segments	2.35 ± 1.060
Percentage of total viability	88.16 ± 6.842

This suggests that despite some non-viable segments, a substantial majority of the myocardium remains viable, which is encouraging for surgical revascularization outcomes. These findings are essential for tailoring individualized treatment strategies and assessing the potential for recovery post-intervention.

## Discussion

In patients with CAD and LV dysfunction, the decision to proceed with CABG is complex and heavily dependent on the assessment of myocardial viability. The relationship between ischemic LV dysfunction and the potential benefit of revascularization has been widely studied, as these patients typically have worse perioperative outcomes compared to those with preserved LV function. The role of advanced imaging techniques, such as LGE on CMR, is crucial in optimizing the selection of candidates for CABG, as it provides vital information about myocardial viability and the likelihood of functional recovery after surgery. The aim of this study was to investigate the association between the ejection fraction (EF%) on echocardiography, myocardial viability assessed by LGE and clinical outcomes in patients with ischemic heart failure treated with CABG. Our results indicated that patients with greater myocardial viability and less non-viable segments demonstrated improved post-surgical outcomes. The observed correlation between myocardial viability and improved outcomes aligns with a previous study conducted by Panza et al., which found that higher viability percentages were associated with reduced morbidity and mortality [18]. Similarly, other studies demonstrated that patients with viable myocardium exhibited better functional recovery post-CABG, suggesting that LGE can serve as a valuable predictor of surgical success [19,20].

The prognostic value of myocardial viability assessments that was observed indicated that patients with higher viability had a significantly lower risk of adverse cardiac events following revascularization [21]. Moreover, recent studies have indicated that patients with significant myocardial viability tend to have a higher quality of life following revascularization [22]. This emphasizes the potential of LGE in not only improving survival rates but also enhancing the overall well-being of patients with ischemic heart failure [23]. The targeted therapies based on LGE results can lead to more tailored and effective treatment plans [24]. The integration of advanced imaging techniques such as LGE-CMR in the preoperative assessment of patients with ischemic LV dysfunction provides critical information that can guide the decision-making process for CABG. Despite the challenges posed by multivessel CAD and comorbidities, careful preoperative evaluation and individualized surgical strategies can optimize outcomes for these high-risk patients. The ongoing development of imaging modalities and surgical techniques continues to improve the prognosis for patients with ischemic LV dysfunction, and future studies should explore the long-term benefits of revascularization in this population.

Limitations

Despite the significant findings in our study, it has its limitations, including a relatively small sample size and short-term follow-up. These limitations may restrict the generalizability of our results. Future studies should aim to address these limitations by conducting larger, multicenter trials that can provide more comprehensive data and validate our findings.

## Conclusions

In conclusion, our study highlights the importance of myocardial viability assessment in predicting clinical outcomes for patients with ischemic heart failure. By integrating LGE findings into clinical practice, healthcare providers may enhance the personalization of treatment strategies and ultimately improve patient care. Further studies should continue to explore the long-term implications of myocardial viability for treatment outcomes and quality of life.
